# Personalized treatment planning with a model of radiation therapy outcomes for use in multiobjective optimization of IMRT plans for prostate cancer

**DOI:** 10.1186/s13014-016-0609-7

**Published:** 2016-03-11

**Authors:** Wade P. Smith, Minsun Kim, Clay Holdsworth, Jay Liao, Mark H. Phillips

**Affiliations:** Department of Radiation Oncology, University of Washington Medical Center, 1959 NE Pacific St, Box 356043, Seattle, 98115 WA USA; Brigham and Women’s Hospital, 75 Francis St., Boston, 02115 MA USA

**Keywords:** IMRT, Multiobjective optimization, Bayesian network, Markov model, Quality of life

## Abstract

**Purpose:**

To build a new treatment planning approach that extends beyond radiation transport and IMRT optimization by modeling the radiation therapy process and prognostic indicators for more outcome-focused decision making.

**Methods:**

An in-house treatment planning system was modified to include multiobjective inverse planning, a probabilistic outcome model, and a multi-attribute decision aid. A genetic algorithm generated a set of plans embodying trade-offs between the separate objectives. An influence diagram network modeled the radiation therapy process of prostate cancer using expert opinion, results of clinical trials, and published research. A Markov model calculated a quality adjusted life expectancy (QALE), which was the endpoint for ranking plans.

**Results:**

The Multiobjective Evolutionary Algorithm (MOEA) was designed to produce an approximation of the Pareto Front representing optimal tradeoffs for IMRT plans. Prognostic information from the dosimetrics of the plans, and from patient-specific clinical variables were combined by the influence diagram. QALEs were calculated for each plan for each set of patient characteristics. Sensitivity analyses were conducted to explore changes in outcomes for variations in patient characteristics and dosimetric variables. The model calculated life expectancies that were in agreement with an independent clinical study.

**Conclusions:**

The radiation therapy model proposed has integrated a number of different physical, biological and clinical models into a more comprehensive model. It illustrates a number of the critical aspects of treatment planning that can be improved and represents a more detailed description of the therapy process. A Markov model was implemented to provide a stronger connection between dosimetric variables and clinical outcomes and could provide a practical, quantitative method for making difficult clinical decisions.

## Introduction

Radiation treatment planning programs (RTP) have historically been models of radiation transport. Over the years, the models have become more sophisticated. The introduction of 3D anatomy and radiation source modeling was an important step forward as was the introduction of more accurate models of radiation transport. Better methods of inspecting the model outputs were constructed, such as isodose lines, dose clouds, and dose-volume histograms.

A significant addition to RTPs are inverse planning algorithms that were introduced in order to manipulate the model variables, namely beamlet intensities, that became available with the introduction of computerized multileaf collimators. These algorithms optimize the beamlet intensities to achieve a set of objectives. While the original intent was to provide a means of solving the highly complex dosimetric beam intensity problem, the algorithms also serve as a de facto decision making process [1–4]. Since the planning objectives are almost always conflicting, it is often impossible to satisfy all the objectives. The most common approach is to combine the multiple objectives into a single linear combination; the coefficients of the separate objectives being related in some subjective manner to their relative values. However, the actual form of the objective function can have a large effect on the solutions which has led to the current practice of trial-and-error in which the weights and function parameters are varied with little or no guidance as to their effect on meeting clinical goals. Recently, there has been published work on automating the inverse planning process. For the most part, these approaches use geometric similarities between a given plan and historical cases to provide a set of objectives for comparison. While this approach can lead to improved clinical efficiencies and may avoid a demonstrably poor plan, it involves a tradeoff between efficiency and a more personalized approach. Our multiobjective optimization seeks to achieve both goals.

Following the introduction of inverse planning algorithms, it was recognized that purely dosimetric objective functions were unable to differentiate satisfactorily between plans that were mathematically equivalent but clinically quite different. This led to the development of radiobiological response objectives that were modeled as functions of the beamlet intensities [5–7]. While these types of objectives are not yet as widespread as the dosimetric ones, numerous studies have demonstrated that the solutions obtained are more likely to satisfy the clinical goals. Regardless of the forms of the objective functions, however, the extensive research that has gone into the form of inverse planning algorithms make it clear that it is difficult to encode clinical reasoning in simple functions of dosimetric variables.

The purpose of this paper is to describe a decision support system for plan selection in radiation therapy. Due to the complexity of each component of this approach we have developed multiple methods independently, and now report on the complete methodology. Our goal was to address three basic weaknesses of the prevailing model of radiation therapy planning. First, we use a multiobjective optimization algorithm rather than the conventional single objective approach. Second, our method explicitly models outcomes of therapy as probabilities, which more accurately reflects the state of our knowledge. Third, the model can handle many different types of variables besides dosimetric variables, and the ultimate objective is related directly to clinical outcomes. We present approaches to all three of these issues, but concentrate on the latter two for reasons of space.

## Methods

**Fig. 1:**
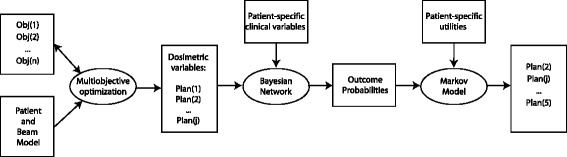
Flow diagram of the new treatment planning model. Objective functions and the patient and beam model are input into a multiobjective optimization algorithm which outputs a set of plans. The set of plan containing the dosimetric variables and patient variables are input into a Bayesian network. The output is a set of probabilities for each of the possible outcomes; these in turn are combined with utilities for these outcomes in a Markov Model in order to rank the plans

Figure 1 illustrates the components of this RTP model. In brief, the anatomy is combined with the radiation transport to devise the basis for optimization of beam fluences. Using a set of decision criteria, a multiobjective optimization algorithm generates a set of plans; each plan in the set achieves the objectives to varying degrees. An influence diagram (see Section below for a more complete definition) models the radiation response of the patient for each plan using both dosimetric and clinical variables and information. Finally, the value (also known as the utility) of each possible outcome is combined with the life expectancy calculated by a Markov Model for that outcome to achieve a ranking of the plans within the set.

The rationale for this particular combination of methods is as follows. Our multiobjective optimization algorithm automatically generates a set of feasible plans that capture a wide range of attitudes to the different possible outcomes. This algorithm is more efficient and more thorough than is possible by an individual planner and by any commercial multiobjective planning system. The production of a set of plans requires the need to decide among them. An influence diagram is an excellent means of representing decision making under uncertainty. It compactly represents the relationships between chance variables and the effects of possible decisions on the probabilities of outcomes and the values of those outcomes. It is more efficient than decision trees and more transparent than neural networks. A Markov model has been used in place of the traditional expected utility since it more accurately reflects the long term consequences of decisions in radiation therapy while at the same time making use of the advantages of utilities. All research was carried out under the guidelines of, and with the approval of, the University of Washington’s Institutional Review Board.

### Multiobjective optimization

The details of our multiobjective optimization algorithm have been described earlier, and in this paper, we provide a conceptual overview [8–10]. The space of all feasible IMRT plans is very large and difficult to search efficiently. We devised a staged approach that tries to balance the size of the search with the time needed to complete the search. The algorithm includes heuristics based on clinical requirements to reduce the time.

At the highest level, we define a set of *decision criteria* which can be of any mathematical form, such as tumor control probability functions or dose-volume metrics. These objectives are assumed to be the most consistent with the clinical goals. At a lower level, we define a set of convex, dosimetric objective functions of the form $f(j) = \sum _{i} \left (d_{i} - d_{j}^{obj}\right)^{2}$, where *i* are the voxels in the organ or target and there are *N* objectives and *j*∈{1,…*N*}. A single objective function is obtained as a linear combination of the dosimetric objectives using a set of weighting parameters, *w*_*j*_; this convex problem is solved deterministically to obtain one optimal plan.

In our algorithm, the plan space is searched by varying the dosimetric objective function parameters, *d*_*obj,j*_, and weighting parameters, *w*_*j*_. This is accomplished by means of an evolutionary algorithm that treats these variables as genes. As each plan is generated using particular values of these parameters, the set of decision criteria functions is applied to the plan, generating a set of decision objective values. Since we view this as a multiobjective decision problem, we use the concept of Pareto optimality to determine whether one plan is superior to another. If all of the decision criteria values of one plan are at least as good as the values of a second plan, and at least one is better, then the first plan *dominates* the second, and the second plan is discarded as being inferior. In many cases, however, some of the decision values of one plan are better and some are worse when compared to those of the second plan. In this case, neither plan dominates and both are kept.

The multiobjective evolutionary algorithm uses Pareto dominant plans to generate “genes” with some probability for mutation. The genetic component is a powerful mathematical method for searching a large space when the objectives are not convex functions. These genes are used to create new plans that are then be compared to the existing population and all inferior plans are eliminated. Using this iterative process of reproduction and natural selection, the population of plans will approach the Pareto Front of optimal plans with respect to the decision criteria. Once the algorithm generates plans that meet the dosimetric constraints defined by the radiation oncologist, randomized trial or QUANTEC [11], it will continue to search for better plans with superior decision objective values as it approaches the Pareto Front. This could mean greater organ sparing and PTV coverage than most plans designed by human planners focused on meeting metrics. The idea is provide a set of plans with a range of tradeoffs which represent the optimal tradeoffs for further evaluation by a radiation oncologist or Bayesian Network that would ultimately result in selection of the single best plan.

#### Influence diagram (Bayesian network)

The second part of the model uses an *influence diagram* to assess the radiation response for a given plan. Bayesian networks (BN) are compact means of representing the joint probabilities of a system. Influence diagrams build on that mathematical framework of probabilistic relationships in order to model decisions under uncertainty. An influence diagram has decision nodes (encapsulating all possible decisions) and utility nodes for representing relative values of different outcomes. Solving an influence diagram for the optimal policy under given conditions uses many of the same mathematical algorithms as a Bayesian network. The output of the model is in terms of probabilities for outcomes of the disease and normal tissues. The goal was to devise a model that reflected our current state of knowledge by using the same sources that are currently used in clinical decision making, namely (a) results from clinical trials, (b) results from the clinical research literature, and (c) expert opinion. Reflecting our current knowledge, the model includes some mechanistic elements as well as some more descriptive aspects depending on how much of the underlying biology is understood.

The Bayesian Network models the process of prognosis [12, 13]. We have previously developed a methodology for combining probabilistic prognostic modeling with multi-attribute decision theory [4, 14], here we present an application to multiobjective optimization with a model that reproduces recent outcome data. In addition, our previous models have been revised and updated based on new insights. Our original development was intended to convey novel approaches in the uses of influence diagrams in clinical decision support systems. These included the use of influence diagrams as a tool for patient-specific decisions, the integration of a range of evidence such as clinical trials and physician judgment, and the use of Markov models in the algorithm for optimal policy selection. Further model development has resulted in the revised model which reproduces the results of an external clinical cohort. We give an overview of the methodology and introduce notation. Within a BN chance nodes represent the variables of the problem and the states of each node represent the values that each variable can obtain, and the probabilities of each state of a variable represents current knowledge. The states can represent the prior probabilities before any evidence is instantiated, posterior probabilities, and certain knowledge. The arrows between the nodes represent probabilistic dependencies between the states of the nodes, and in a BN the arrows are said to be drawn from parent nodes to child nodes. Conditional probability tables provide the quantitative connections between the parents and children. The probability that X will take on the value *x*_*i*_ given that the parent states are *a*_*j*_ and *b*_*k*_ is *P*(*X*=*x*_*i*_|*A*=*a*_*j*_,*B*=*b*_*k*_). The BN was implemented using the software package Hugin (Hugin Expert A/S, Aalborg, Denmark.)

The process for creating an BN involves writing down the variables which affect the outcome of the treatment, and drawing links between all of those variables which influence one another. Each variable is characterized by a set of possible states and each connection symbolizes a conditional probability. Modeling methods were employed in order to reduce the number of conditional probabilities required [13]. For clarity, we introduce the notation that the node variables are denoted using the SMALL CAPS font. Variables in the BN are prefixed by BN: and those in the Markov model (see below) by the prefix MM:.

**Fig. 2:**
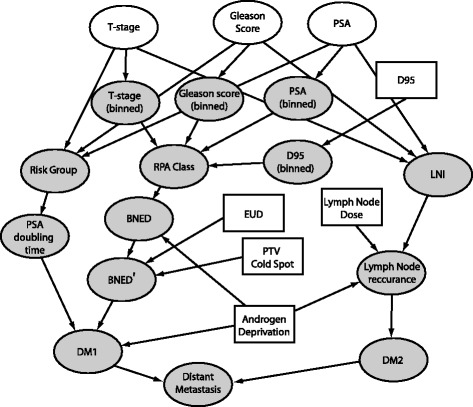
Bayesian network representing response to radiation therapy of the prostate and regional lymph nodes. White ovals denote variables with patient-specific clinical evidence; gray ovals denote chance variables; rectangular nodes are decision variables

Our BN for prostate cancer prognosis following radiotherapy consists of three separate networks dealing with tumor control, rectal toxicity, and bladder toxicity. The network for disease control is depicted in Fig. 2. The network calculates the three probabilities which are used by the Markov model: (a) biochemical control following radiotherapy, (b) freedom from distant metastasis for patients with biochemical control, (c) freedom from distant metastases for patients with biochemical relapse. Distant metastases are modeled as spreading from the primary site and also as progression of initially present occult metastases. We also explore another pathway which models distant metastases as progression from regional involvement of the lymph nodes. We explore different assumptions about the possible cure rate of this pathway.

Patients with lymph node involvement that is detectable at the time of disease staging have a different set of treatment options, and we do not model this cohort. The likelihood that our patient cohort has occult lymph node involvement is predicted by their pathological staging [15]. The node BN:LYMPH NODE INVOLVEMENT represents the probability that lymph nodes are initially involved. As an example of the type of decision that may be explored by this type of model we explore the outcomes of lymph node treatment with whole-pelvic radiation.

PSA levels typically decrease following radiotherapy treatment, and patients are said to have biochemical no-evidence of disease (BNED) if their PSA levels remain low. BNED failures arise from increased PSA production at either the location of the primary disease or from distant metastases. The conditional probabilities for the node BN:BNED, in the absence of androgen deprivation, come from a retrospective clinical analysis [16]. This study used recursive partitioning analysis (RPA) and grouped patients into one of four RPA classes with 5-year BNED control rates. The doses used in this study (67 – 81 Gy) are from the pre-IMRT era, as long-term survival evidence accumulates it is a simple process to revise the probability table of this node.

Many patients undergoing external beam radiotherapy for prostate cancer also receive some form of androgen deprivation therapy (ADT). The node BN:ANDROGEN DEPRIVATION contains three states corresponding to no ADT, 3-months of ADT, or 6-months of ADT. A large, controlled, randomized trial [17] compared two different short-term ADT treatment schedules to no-ADT for patients receiving an average of 66 Gy to the prostate and finds 5-year BNED failure rates of 28 % for no ADT, 17 % for 3-months of ADT, and 12 % for 6-months of ADT. We selected this study to modify the predictions from the study in Horwitz et al. [16] because it is one of the few studies to include a cohort receiving no ADT.

An example of the expert-experience driven decision making occurs in the BN:BNED’ node of the network. The model includes two IMRT plan parameters, the volume of the planning target volume (PTV) which receives a dose lower than the prescription dose (BN:COLD SPOT), and the equivalent uniform dose (EUD)[18] which is defined as the uniform dose which would result in the same amount of cell killing in the PTV as the planned non-uniform dose-distribution. The conditional probability table for BN:BNED’ reflects the belief that the probability of tumor control is increased by larger values of the equivalent uniform dose and smaller volumes which are below the prescription dose within the PTV.

One pathway for development of distant metastases is a post-treatment rise in PSA levels, modeled in the node BN:DM1. The prognosis for patients with a rising PSA level is not well defined. One indicator is the post-treatment PSA doubling time. At the time of radiotherapy plan selection, however, the only information available is pretreatment pathological staging. This staging can be used to predict the post-treatment PSA doubling time for patients who fail, using T-stage, Gleason score, and (pre-treatment) PSA level [19]. A rising PSA level is not a perfect predictor for the development distant metastases, of course, as some patients with a steady PSA develop distant metastases and some patients with a rising PSA never develop distant metastases. The sensitivity and specificity for the ASTRO definition [20] of biochemical failure are 73 % and 76 %, respectively [21], and have been incorporated into the node BN:DM1.

Another pathway for the development of metastases included in the model is occult lymph node involvement, modeled in the node BN:DM2, and BN:DISTANT METASTASIS is modeled as an “or” node which is positive if either pathway is positive. We include both pathways in this outcome model to order to explore whether treatment of the lymph nodes – given different possibilities of occult disease, patient preferences, and physician beliefs – is worth additional treatment toxicity.

**Fig. 3:**
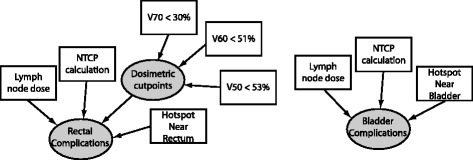
Bayesian network representing normal tissue response to radiation therapy for cancer therapy. Bladder and rectum were the two normal tissues selected

The Bayesian Network for predicting toxicity is depicted in Fig. 3. In prostate cancer, the rectum and the bladder are the two primary organs at risk for complication. The probability for developing toxicity is modeled as a combination of a normal tissue complication probability (NTCP) computation, a consideration of hotspots in regions near the organ at risk (OAR) at the time of imaging, and three dosimetric objectives which have been found to be predictive in a retrospective analysis [22].

We calculate the NTCP with the Lyman-Kutcher method [23–25]. The parameters used in our calculation are averages from three studies [26–28]. Bladder complications are a less likely side effect of radiotherapy, and only one study was found fitting bladder complications to the Lyman-Kutcher model [29]. Both studies use endpoints similar to the descriptions presented to patients for utility determination (discussed below). The NTCP model includes dose-volume information based on an organ volume as visualized by a CT scan. This static model does not account for set-up error or organ motion within the patient. A hotspot near the rectum, for example, is subject to some uncertainty in its daily location, and some physicians prefer to keep the hotpot a distance away from the rectum for this reason. The preference for plans without a hotspot nearby reflects the physician belief that the outcome for such a plan may be better than for a plan with a hotspot nearby. We modeled this by increasing or decreasing the probability of a complication as a function of whether hotspots – defined as a dose higher than TD50 – were nearer or further from the organ. Such an approach was based on observing clinical judgments and recent results regarding rectal complications [30].

#### Markov cohort simulation

The Markov Model for calculating the Quality Adjusted Life Expectancy (QALE) goes beyond the Bayesian Network by calculating the longterm effects and also includes the values associated with the expected outcomes. The probabilities calculated by the Bayesian Network are used an input to a Markov Cohort Simulation that is used to track subsequent health state evolution. A Markov Cohort Simulation follows some fixed number of simulated patients through a state transition model, tracking the amount of time spent in each state. The total amount of time spent in each state divided by the number of patients in the simulation yields the life expectancy. The amount of time spent in each state is weighted by a utility of that state to calculate the QALE. The QALE is then used to rank the plans from best to worst.

**Fig. 4:**
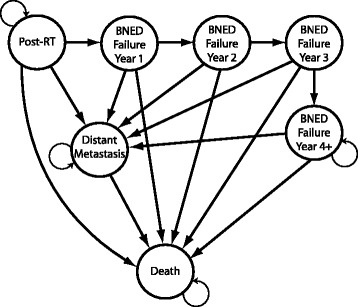
The Markov model used to calculate the quality-adjusted life expectancy of a patient using transition probabilities from the Bayesian network, and life expectancy data for a healthy individual

For our Markov Model (see Fig. 4) many of the transition probabilities are time-dependent. The probability to die of natural causes is age-dependent and is taken from Social Security Administration Life Expectancy tables [31]. Distant metastases develop most commonly after it is discovered that a patient has a rising PSA level, but they are also discovered in the absence of PSA failure. The Bayesian Network calculates the probabilities for development of distant metastases through both mechanisms, in the node BN:DISTANT METASTASIS with different evidence (YES or NO) entered into BN:PSA FAILURE.

A Markov Cohort simulation handles a certain time-dependent transition probability by changing the transition probability each time the model is updated. This method is used to handle probabilities which are a function of how much time has passed since the initiation of the model. Two examples of this type of time-dependent transition probability are the probability of death from natural causes, which increases yearly as the patient ages, and the probability to develop PSA failure, which is fixed for the first 5 years after treatment and then set to zero.

Time-dependent transition probabilities which are not a function of the model’s initial state are handled by tunnel states. These states do not have a transition probability back into themselves, and are used to model risks that develop sometime after the first cycle. In our case the probability to develop distant metastases in the first 3 years after developing a rising PSA is greater than in subsequent years [19]. We model this situation by dividing the biochemical failure period into an early, ≤ 3 years, stage with a high probability for development of DM, and a late, ≥4 stage. Both stages assume a constant rate model, each with a different rate. The rate in the later stage is set to one third that of the initial rate. Once the model reaches 15 years post-RT the probability to transition into the Distant Metastasis state is turned off.

*P*(*DM*|*PSAControl*=*yes*) determines the transition probability from POST-RT to DISTANT METASTASIS and is the probability that a patient will develop DM without first developing a rising PSA. *P*(*DM*|*PSAControl*=*no*) is the probability that a patient with biochemical failure will develop distant metastases, and determines the transition probability from the tunnel states BIOCHEMICAL FAILURE, YEAR 1,2, OR 3 to DISTANT METASTASIS. The transition probability to develop DM without biochemical failure is turned off after year 5. The cycle-length is 1 year. The model runs from the time of RT until the patient would be 119 years old, which is the oldest age for which the SSA has life expectancy data. The median survival time for patients who develop DM is 19 months [32].

The prognostic probabilities from the BN are cumulative probabilities for certain events at certain time points, defined by clinical studies. The probability calculated in BN:BNED, for example, is the probability to have biological no evidence of disease at *t*=5 years after the beginning of RT. These prognostic, cumulative probabilities are transformed into annual transition probabilities for the MM. Each annual transition probability is calculated by assuming a constant annual rate, *r*_*a*_, related to the cumulative probability of an event from the BN as $p_{c} = 1 - e^{-r_{a}t}\phantom {\dot {i}\!}$ where *p*_*c*_ is a cumulative probability from the BN, and the subscript *a* denotes annual. The annual transition probability *p*_*a*_ for the MM is then simply $\phantom {\dot {i}\!}p_{a} = 1 - e^{-r_{a}}$.

We consider late complications only and approximate each complication as occurring at the end of the second year of the simulation and persisting throughout the lifetime of the patient. Complications are assumed to affect each state of the Markov model with an equal probability, and they do not affect transition probabilities into other Markov states. The effects of complications are neglected for patients with metastatic cancer since the utility for metastatic disease is much lower than for treatment side effects for prostate cancer treated with radiotherapy.

The utilities for health states used in the QALE calculation were obtained from reference [33]. Three sets of values were used: (1) the average values of the utilities reported, (2) utilities which were two standard deviations below the average, chosen to represent individuals who are strongly averse to complications and (3) utilities of 1.0 to reflect the preference for maximizing survival regardless of cost. The cohort simulation is coded in the programming language LISP [34].

In addition to examining decisions for specific patient characteristics we performed a continuous sensitivity and threshold analysis using linear regression metamodeling. First, a probabilistic sensitivity analysis was performed on the model, whereby the the probabilities and and utilities in the model are represented by a normal distribution which has been truncated to accurately represent physical values – for example between 0 and 1 for a transition probability. 10,000 values are chosen at random from each distribution which represent 10,000 separate patient cohorts [35]. The output of this model is QALY values for the different cohorts, and a linear regression model – a metamodel – is fit to the model inputs and outcomes and used to perform sensitivity analysis [36].

## Results

In this initial report of our RTP model we have focused on investigating the structure and accuracy of the system. To this end, we present several different results. The first was to perform a sensitivity study of the life expectancy as a function of dose. The second was to compare the calculations of survival and toxicity with an independent study. The third was to calculate the outcomes for a given plan for patients with a range of different characteristics. The fourth was to investigate the output of the model for a range of plans produced by the multiobjective optimization procedure described above.

Unless otherwise stated, “high risk” patients were modeled as a T2c, pre-treatment PSA = 25 ng/ml, Gleason sum = 8, and “intermediate risk” as T2a, 15 ng/ml, 7. Risk group classifications are according to the National Comprehensive Cancer Network practice guidelines for recurrence risk groups (www.nccn.org).

Sensitivity study: Table 1 is a sensitivity study for the responses of the LE and QALE from increasing the tumor dose for intermediate and high risk patient populations. Clinical trials for 3D-conformal treatments have shown little evidence of improved outcomes by raising the dose of the intermediate risk patient above 74 Gy or for treating a high risk patient above 78 Gy [16]. For the change in dose for the intermediate risk patient, the life expectancy increases 8.4 months and the QALE increases by 4.8 months. For the high risk patient, the corresponding increases are 6.0 and 3.6 months, respectively. The QALE increases more for the intermediate risk patient than for the high risk patient for the same absolute change in dose because the lower dose results in fewer complications for the intermediate risk. In both cases, the higher dose is worth the risk.

**Table 1:** Life expectancy (LE) [years] and quality-adjusted LE (QALE) [years] for an intermediate risk and a high risk 60 year old patient for different values of *D*_95_ [Gy]

Risk category	Dose	LE	QALE
Intermediate	71	17.7	12.2
	74	18.4	12.6
High	75	15.9	10.8
	78	16.4	11.1

Survival and toxicity calculations: Modeling a clinical study from data presented in the literature can be difficult if the patient data is presented in summary form only. Data sets with patient specific dosimetry, age, clinical scoring, treatment information, and outcome are rarely reported. Modeling limitations aside, however, it is instructive to see if a modeled cohort which approximates the average characteristics of a clinical cohort performs similarly to the clinical cohort.

**Fig. 5:**
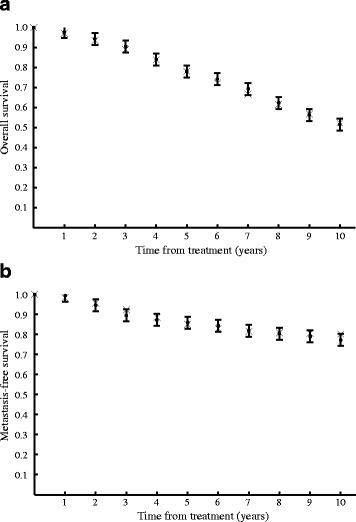
**a** Overall survival and **b** metastasis-free survival from a recent randomised, prospective clinical trial [37] (solid circles) compared with predictions from the model (x marks)

Figure 5 presents comparisons of our model predictions for overall survival and metastasis-free survival with a randomized, prospective clinical trial, RTOG 92-02 [37], which is different than the studies from which we obtained our modeling parameters (see above). This clinical trial studied the difference between 4-months of ADT and a 2 year course of ADT on high risk (T2c-T4) prostate cancer patients. We compare our predictions for 3-months of ADT with their short-term ADT results. The clinical results reported a 95 % confidence interval at year 10 of ±3 *%*; we apply the same interval as an approximation to the entire time course. The greatest source of uncertainty in our model is the BNED control rate, and we performed a sensitivity analysis by varying the control rate for each RPA class between the control rates of the closest other RPA classes. This results in uncertainties at year 10 in our predictions for overall survival of 2 %, and metastasis-free survival of 2.5 %. We do not plot the model uncertainties in the figure for ease of reading. The model predictions for overall survival and metastasis-free survival all fall within the confidence interval of the clinical data.

Toxicity data for prostate cancer is more limited than survival data since clinical trials are powered to see effect sizes in survival, and toxicity rates in prostate cancer are generally low. Furthermore, bladder toxicity rates for prostate cancer are notoriously inconsistent between studies, due to sparse data and to the fact that bladder motion is often not considered [38]. Finally, published studies rarely – if ever – make available the dose distributions that are required for an NTCP calculation. The application of the model to multiobjective optimization (see below) is an intended use of the model for patient-specific decisions, where dosimetric data for multiple treatment plans is available. For the sensitivity analysis performed here the baseline grade 2 or greater toxicity predictions for an intermediate stage patient receiving whole pelvic radiotherapy are 15 and 16 % for bowel and bladder, respectively, compared with RTOG 92-02 [37] which reports an overall incidence of grade 2 or greater bowel and bladder toxicities of 16 and 18 %, respectively.

Patient characteristic study: Table 2 compares QALEs for different sets of patients treated with whole pelvic radiotherapy (WPRT) or prostate only RT (PORT) under different assumptions for the curative effect of lymph node irradiation from WPRT. In the absence of definitive data regarding the curative effects of RT for lymph node disease, the decision about when to treat lymph nodes is affected by a physician’s belief in the cure rate. We examine two cure rates, 20 and 65 %, to illustrate how personal physician beliefs can be encoded by the BN. Those who believe that lymph node irradiation is not curative, of course, would not elect to use WPRT. One argument against treating with elective lymph node irradiation is that it is generally believed to be associated with a higher rate of side effects, although definitive data on complications with WPRT does not exist. For this study we modeled WPRT as increasing the likelihood complications by 8 % in each OAR.

**Table 2:** Quality Adjusted Life Expectancies [years] for different types of patients, and two different physician beliefs in the curative ability of lymph node irradiation

Patient preference	Age	Risk group	PO ^*a*^	WP$^{b}_{65\,\%}$	WP$^{c}_{20\,\%}$	Optimal choice ^*d*^
Min-complications	60	intermediate	12.0	11.4	11.0	PO
	60	high	9.4	9.9	8.8	MD
	75	intermediate	7.1	6.7	6.4	PO
	75	high	5.8	5.9	5.3	MD
Population average	60	intermediate	12.3	12.6	12.1	MD
	60	high	9.8	11.1	10.0	WP
	75	intermediate	7.2	7.3	7.1	MD
	75	high	6.0	6.6	6.1	WP
Max life	60	intermediate	12.5	13.1	12.7	WP
	60	high	9.9	11.7	10.5	WP
	75	intermediate	7.3	7.7	7.4	WP
	75	high	6.1	7.0	6.3	WP

^*a*^PO = prostate-only RT

^*b*^ WP_65 *%*_= whole pelvic RT assuming a 65% cure rate for occult lymph node disease

^*c*^ WP_20 *%*_= whole pelvic RT assuming a 20% cure rate for occult lymph node disease

^*d*^MD = optimal choice depends upon physician belief in efficacy of nodal irradiation

Patients are stratified by age, disease risk group and attitude towards the balance between quantity and quality of life

The optimal choice for each patient is listed in the final column of Table 2. The patient “max life” makes all decisions in order to maximize life expectancy, regardless of the severity of the complication outcome, therefore all complication utilities are set to 1.0. This patient would elect any treatment believed to offer a higher cure rate, and whole-pelvic therapy is the optimal choice predicted by the model for this patient set, regardless of age, risk group, or the efficacy of whole pelvic radiotherapy (as long as it is greater than zero). In general, whenever a single factor is the deciding factor a decision model is not necessary, in this case only an outcome model is needed in order to predict cure rates. A recent study of prostate cancer complication state utilities found that 17 % of those surveyed were in this category [39]. For other attitudes towards health states, the remainder of the patients, the situation is more complex.

For patients with population-average attitudes towards health states and intermediate to high risk disease, whole-pelvic radiotherapy would be the optimal action, since their high probability of lymph node involvement means that the additional cure rate offered by WPRT more than balances the additional toxicities. In our model the BN calculates a 26 % chance of lymph node involvement (BN:LNI) for the high risk sample patients, and a 10 % chance for the intermediate risk patients. Note that the recommendation for high-risk patients is independent of the physician belief in the efficacy of WPRT within the boundaries studied. The situation is more complex for intermediate-risk patients with population average attitudes towards health states since the QALE result depends on the physician’s belief in the efficacy of WPRT. If WPRT is effective in 65 % of the cases, then WPRT should be elected, if WPRT is effective in only 20 % of the cases, then prostate-only treatment should be elected. For a 60 year old intermediate risk prostate patient with average attitudes towards health states prostate-only radiotherapy and whole-pelvic radiotherapy offer the same QALE if whole-pelvic radiotherapy is curative in 40 % of cases. In this regard the model calls attention to the fact that better data as to the efficacy of lymph node irradiation, both in regards to the absolute value and uncertainty of the control rate, is needed.

In order to provide recommendations for patients who are highly averse to toxicities, the calculation was repeated with utilities for toxicity states which were two standard deviations below the population average, labeled “min-complications” in Table 2. For intermediate risk patients in this group, prostate-only therapy offers the highest QALE, regardless of patient age, or the efficacy of lymph-node irradiation, within the boundaries studied. The situation is more complex for high-risk patient who are complication averse, as their optimal choice depends on the physician belief in WPRT, a similar situation to the intermediate risk patients with average attitudes towards toxicity discussed above.

**Fig. 6:**
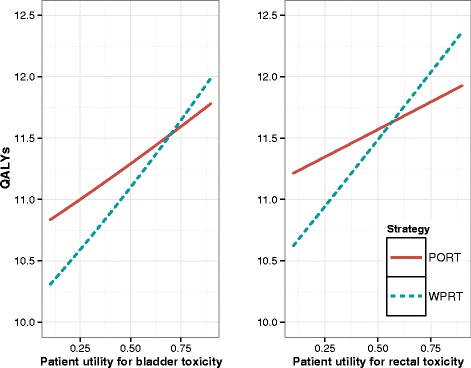
Sensitivity analysis for patient utilities for bladder and rectal toxicity

The results of one-way continuous sensitivity analysis for two patient utilities for toxicities is presented in Fig. 6. QALYs are plotted on the vertical axis for two strategies, PORT and WPRT, with the utility value on the horizontal axis. The rest of the parameters are held constant, and are for a 60 year old, PSA 22 ng/ml, Gleason 4 + 3, T2a patient, with a WPRT control rate of 50 %. The steeper slope of the WPRT line reflects the fact that the model predicts a higher incidence of toxicities for WPRT, so varying the utility has a larger affect on QALYs. In both cases a patient who assigns a low utility to a toxicity state (complication-averse) is offered higher QALYs by PORT due to its lower toxicity rate, and a patient who assigns a higher utility to a toxicity state is offered higher QALYs by WPRT from its higher disease control rate.

**Fig. 7:**
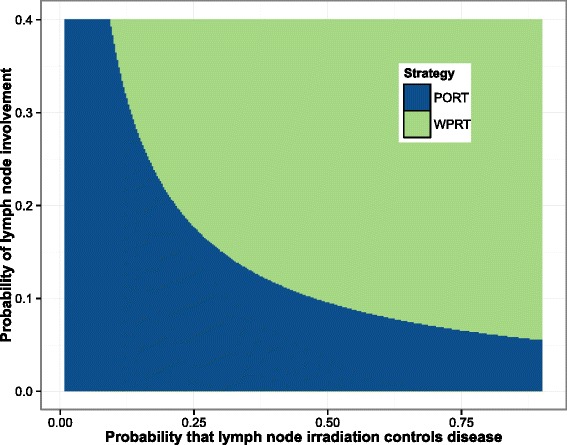
Two-way threshold analysis for the probability of lymph node involvement and the control rate of lymph node irradiation. The strategy that offers the most QALYs for the pair of parameters is the dominant strategy. WPRT is dominant strategy in the blue region of the plot, and PORT is the dominant strategy in the green region

The results of a two-way continuous threshold analysis are presented in Fig. 7. The probability of lymph node involvement is plotted on vertical axis, with the probability that whole-pelvic radiation controls disease plotted on the horizontal axis. Other model parameters are held constant for the same patient used in the previous example, using population average values from the literature for toxicity states [33]. The different colors depict which strategy is dominant for different combinations of model parameters. The region in blue offers the highest QALYs for PORT for this patient, and the region in green is the dominant strategy for WPRT. It is common practice for physicians to offer lymph node irradiation to patients with lymph node involvement above a particular threshold, this analysis demonstrates that the physician’s belief in the efficacy of WPRT should be taken into account when setting this threshold.

**Fig. 8:**
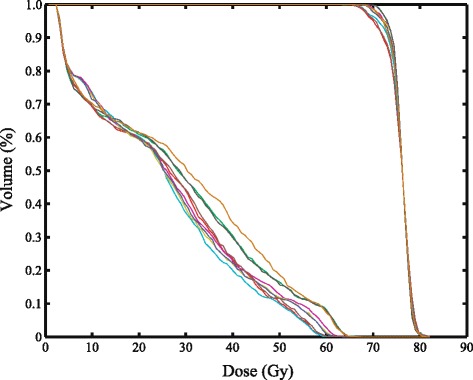
Dose volume histograms for the prostate and the rectum for 10 Pareto optimal plans generated by the multiobjective optimization routine

Multiobjective optimization: The multiobjective algorithm produced ten plans. Figure 8 shows the prostate and rectum DVHs for the ten plans. The target objective was 76 Gy to ≥95 % of the volume. The QALEs were calculated for a high risk and an intermediate risk 60 year old patient with average attitudes towards risk. For the high risk patient, the QALEs ranged from 10.6 – 11.6 years; for the intermediate risk patient they range from 12.6 – 12.7. The smaller range for the intermediate risk patients stems from the fact that *D*_95_ for these plans are all at or above the level found to be beneficial for intermediate risk patients and therefore there was little variation between plans in the effects on control rates. The range is due to the differences in EUD, the size of the cold spot, and the probability of complications induced.

## Discussion

The development of the model described above resulted from the recognition that a number of different aspects of current radiation therapy models were less than optimal and needed to be improved if the field is to deal effectively with the increased complexity of current and future practice. This complexity arises from the need to include large numbers of variables in the optimization algorithm in an effort to steer the result to a clinically desirable plan. It also arises because the inverse planning algorithm has essentially been transformed into a decision making algorithm that must deal with the trade-offs inherent in the problem. The multiobjective approach we describe is a more thorough approach to searching this high-dimensional space and provides a better method for viewing the trade-offs than our current trial-and-error methods which are sensitive to the planner’s experience and preferences.

In selecting a method for improving the modeling of radiation therapy, we were guided by the desire to incorporate a number of key attributes of the problem in the model. The first was to deal explicitly with the probabilistic nature of our knowledge between the parameters of therapy and the outcomes. The second was to incorporate the diverse types of information that influence the process of selecting between competing plans. Finally, the model had to be flexible enough to adjust easily to the introduction of new variables, e.g. biomolecular or genetic information, and updated data regarding outcomes, such as from recent clinical trials.

The current model builds on our previous work [4, 14, 40]. In that work, it was shown that a Bayesian network is an appropriate mathematical framework for dealing with the model attributes of interest. Comparisons of the current prostate cancer model with that described in our earlier work provide proof of the ability to modify and add to the model. Important extensions were to represent the clinical reality by coupling the time-dependent probabilities computed by the Bayesian Network with a relevant clinical endpoint, namely QALE, through the means of a Markov model and the inclusion of the effect of androgen-deprivation therapy.

It should also be noted that BNs have another characteristic that makes them an excellent choice as a medical decision aid. The ability to inspect each node and its role in the final probabilities makes it easy to isolate the most critical variables and to focus efforts at improvements on the appropriate elements of the model. This was seen clearly in the comparison of multiobjective plans described above. For high risk patients, significant differences in QALE were calculated; for intermediate risk patients all QALEs were very similar. Inspecting the nodes of the model, it was determined that for intermediate risk patients, the dose chosen was high enough that small differences in dose had negligible effect on the outcome whereas for high risk patients, the differences were notable. We repeated the experiment with a target dose of 72 Gy and the results were reversed so that the plan differences for the high risk patient were negligible because the dose was too low to have a significant impact on high risk patient survival. If these results do not match expected outcomes, it is clear how to modify the model to account for the changes. This example also illustrates nicely the effect of having mixed patient populations in clinical studies. If a clinical study included patients in both risk groups, it is clear how the effects of different doses would be washed out.

In the biological realm, there exist models of the effects of fractionation that are quite detailed with respect to the biological mechanisms. While the predictive power of these models is questioned by some, they are widely used for calculating the dose required to maintain the same biological effect when the fractionation schedule is altered. Another active area of modeling is the effect of dose at both the cellular and organ level. Swanson et al. [41] have published a model describing the migration of glioblastoma cells in the brain, thereby providing a possible explanation of the response of these types of tumors to radiation therapy. Blijlevens et al. [42] published a detailed model of the biological mechanisms involved in mucositis. Benson et al. have developed a Markov model to calculate the probabilities of lymph node involvement in head and neck cancer [43]. To date, however, such models are rare and have not made their way into regular clinical practice, although our model could include their potential effects on clinical decision-making.

As described above, there are logistic regression models for tumor control and normal tissue complications which are used in inverse planning. One publication [44] examines four different statistical logistic models for predicting radiation-induced pneumonitis. This is an interesting paper in that each of the four models were derived from the same data set. The model-building methods for each resulted in different sets of variables being included. This paper demonstrated that even in this limited realm of a given patient data set better predictions could be obtained by combining the models together, an important feature of our model. This paper also showed that different factors modified a basic logistic response curve by shifting the curve along the dose axis. We have used a similar procedure in combining effects in our BN.

The role of mathematical models in predicting outcomes of medical procedures is increasing in most areas of medicine. Recent emphasis on evidence-based medicine is likely to increase that role if the models are based on clinical data. The model described in this paper, while designed to select an IMRT plan most appropriate for given plans, can easily be expanded to include more basic choices in the types of therapies as well. In the context of public health decisions, predictions of outcomes such as QALEs are even more appropriate. We consider this to be a “fractal-like” model, in that the conditional probability tables for any node can be expanded to include another model that uses more detailed mechanisms to provide probability values.

As the model becomes further refined it may be possible to offer treatment guidelines for questions involving elective therapies, as demonstrated in this work, by the inclusion of the different possible cure rates of lymph-node irradiation. In this context, modeling can identify groups of patients grouped by similar characteristics such as age, clinical staging, personal preferences, and achievable planning (DVH) goals given patient geometry to recommend an approach for an individual which offers an optimal quality-adjusted life expectancy. This approach is similar to the current standard of care where treatment guidelines are based primarily, if not exclusively, on clinical staging.

The next step in this project is to conduct a systematic comparison between the rankings of a set of plans by radiation oncologists with the rankings produced by this model. The purpose will be to better understand the variables used for clinical decision making and to fine-tune the model to produce similar results. This process will also help to determine the extent to which different physicians can agree on plans and the reasons for their agreement/disagreement.

## Conclusion

A model of radiation therapy outcomes was described in the context of multiobjective optimization for IMRT. The model calculated life expectancies that were in good agreement with an independent clinical study. In the context of current model-building in radiation oncology, this model is much more comprehensive. In particular, this is the first model applied to the results of a multiobjective optimization which utilizes an evolutionary algorithm. Other models, such as NTCP and EUD, are useful in providing key probabilities in our model. The method is personalized in two senses, one, the multiobjective optimization algorithm works on individual patient anatomy, and two, individual patient preferences can be used in the Markov Model to optimize an individual’s QALE. Future work will focus on tuning the model to obtain agreement with expert opinion, and also will include modeling other tumor sites.
